# Effects of mother–infant skin‐to‐skin contact on mother–infant relationship and maternal psychology feelings: A qualitative study

**DOI:** 10.1002/nop2.2181

**Published:** 2024-06-21

**Authors:** Xiaoyan Feng, Yuqing Zhang

**Affiliations:** ^1^ Department of Obstetrics The First Affiliated Hospital of Chongqing Medical University Chongqing China; ^2^ Nursing Department The First Affiliated Hospital of Chongqing Medical University Chongqing China; ^3^ Research Center for Adolescent Personality and Health, Institute of Psychology Chinese Academy of Sciences Beijing China

**Keywords:** maternal psychology feelings, mother–infant relationship, mother–infant skin‐to‐skin contact, qualitative study

## Abstract

**Aims:**

To explore the effects of mother‐infant skin‐to‐skin contact on mother‐infant relationship and maternal psychology feelings.

**Design:**

An exploratory qualitative research design using semi‐structured interviews.

**Methods:**

A total of 64 mother‐infant pairs who met the inclusion criteria were selected as the experimental subjects to receive early and continuous intervention of mother‐infant skin‐to‐skin contact (SSC). On this basis, the qualitative research method of procedural grounded theory was used to conduct semi‐structured interviews with 18 puerperas before discharge from the hospital; the three‐level coding method of procedural grounded theory and Graneheim & Lundman qualitative content analysis method were combined to conductinterview content analysis in Nvivo 12 software, so as to extractcore categories and condense the theme.

**Results:**

(1) The data were coded to extract five core categories, namely, birth experience, role transition, contact perception, mother‐infant connection and parental efficacy; (2) there were statistically significant differences in the number of coding reference points in five nodes before and after SSC, that is, mothers' positive feelings, newborns' physical characteristics noticed by their mothers, mother‐infant connection, role transition and birth experience. The number of coding reference points after SSC was statistically significant greater than before SSC; (3) The coding interview results showed that SSC could promote the sense of happiness in nurturing.

## 1 INTRODUCTION

Mother–infant skin‐to‐skin contact (SSC) refers to placing an unwrapped newborn on the mother's bare chest and abdomen for direct contact after birth, without separating the skin with clothing or blankets (Widström et al., 2019). As a basic and inexpensive early childhood health intervention, SSC has many benefits for mothers and infants (Xie & Liu, 2021). One study showed that SSC is beneficial in maintaining the stability of neonatal body temperature and blood glucose, helping the newborn adapt to the extrauterine environment, improving the breastfeeding self‐efficacy of puerpera, increasing the exclusive breastfeeding rate and promoting the duration of breastfeeding (Karimi et al., 2014, 2019, 2020), and enhances the secretion of endogenous oxytocin to shorten the third stage of labour and reduce postpartum haemorrhage (Karimi et al., 2019; Khadivzadeh et al., 2018). The Baby‐Friendly Hospital Initiative requires that the infant should have SSC with the mother immediately after birth for at least 1 h (Gomez‐Pomar & Blubaugh, 2018). However, in clinical practice, SSC is not performed in a timely manner and the contact time is inadequate in healthcare settings due to safety and feasibility considerations (Wu et al., 2019). In addition, as pointed out by Xie and Liu (2021), most studies in China focus on the effects of SSC on the physiological aspects such as infant feeding, sleep and crying and few studies focus on the effects of SSC on maternal psychological feelings and mother–infant relationship. Based on the continuous implementation of early SSC for more than 1 h, this study used semi‐structured interviews to explore the psychological status of mothers with SSC and the mother–infant relationship, and tried to propose a theoretical model to provide a basis for further implementation of SSC in maternal and child healthcare institutions in the future, now it is reported as follows. This research has been approved by Chongqing Key Specialized Construction ‘Clinical Nursing’ Boutique Construction Project 0203 [2023] No. 47202336.

## 2 THE STUDY

### 2.1 Aim(s) and objective

To explore the effects of mother–infant skin‐to‐skin contact on mother–infant relationship and maternal psychology feelings.

### 2.2 Technical terminology used to describe the aim

To explore the positive effects of SSC from the perspective of mother–child relationship and maternal psychological feelings using qualitative study methods.

## 3 METHODS

### 3.1 Design

**FIGURE 1:**
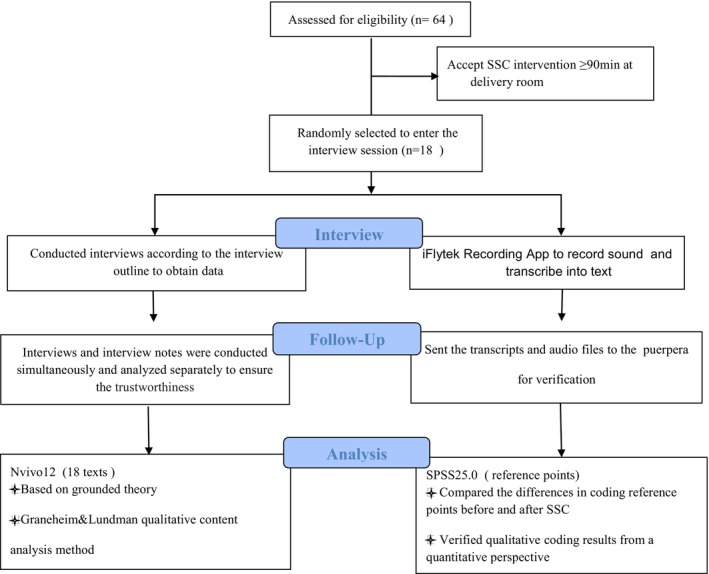
The study flow path.

Based on the randomized controlled experiment, a qualitative study with semi‐structured interviews was conducted, as shown in Figure 1.

### 3.2 Theoretical framework

Skin‐to‐skin contact may play a role in regulating hormone levels in mothers and infants. Based on attachment theory and good enough mother theory, explore relationships and psychological feelings.

### 3.3 Sampling and sample size

From March to July 2022, a total of 64 mother–infant pairs who gave birth naturally in the obstetrics department of a third‐class A hospital in Chongqing, China, were selected as study participants by the random sampling method, all participants underwent SSC intervention and 18 puerperae were randomly selected according to the principle of voluntary participation in interviews. Sample saturation was terminated based on the criterion of no new interview information appearing again.

### 3.4 Inclusion and exclusion criteria

Inclusion criteria: (1) puerpera with gestational age greater than 37 weeks; (2) puerpera without severe pregnancy complications; (3) puerpera without mental and communication disorders; (4) puerpera who were willing to accept SSC; (5) puerpera without postpartum complications and infant complications; (6) the newborns had a weight greater than or equal to 2500 g and their 5‐min Apgar scores were 10 points (among the infants with 1‐min Apgar score of <10 points after birth, those with 5‐min Apgar score of 10 points after initial resuscitation could undergo SSC intervention).

Exclusion criteria: (1) those who were unconscious and unable to cooperate after infant delivery; (2) mother–infant separation; and (3) the newborns with a weight of less than 2500 g, whose status was not allowed for SSC intervention after evaluation. The above inclusion criteria applied to mothers and infants who participated in the experiment.

### 3.5 Population and SAMPLE

The interviewees were selected from the experimental participants and also met the criteria; a serial number was used instead of the name (N). Eighteen pregnant women were interviewed, accounting for 28% of the total participants, the mean age was (29.56 ± 3.24) years old, multipara accounted for 16.7% and first birth accounted for 83.3%. For the maternal education information, senior high school was 5.6%, junior college accounted for 16.7%, undergraduate was 55.5% and the proportion of master was 22.2%. For the newborn sex information, 66.7% were boys and 33.3% were girls. The basic information of 18 interviewees was as shown in Table 1.

**TABLE 1:** Basic information of interviewees (*N* = 18).

No.	Age	Occupation	Education level	Parity	Neonate gender
N1	32	Enterprise staff	Master	First child	Male
N2	33	Enterprise staff	Master	First child	Male
N3	30	Enterprise staff	Undergraduate	First child	Male
N4	24	Flight attendant	Junior college	First child	Male
N5	28	Institution staff	Undergraduate	First child	Male
N6	30	Enterprise staff	Junior college	Second child	Male
N7	31	Stay‐at‐home moms	Senior high school	Third child	Female
N8	31	Enterprise staff	Undergraduate	First child	Female
N9	29	Hospital finance officer	Undergraduate	First child	Male
N10	24	Enterprise staff	Undergraduate	First child	Female
N11	32	Enterprise staff	Undergraduate	First child	Male
N12	28	Teacher	Undergraduate	First child	Male
N13	34	Nurse	Undergraduate	Second child	Female
N14	26	Stay‐at‐home moms	Junior college	First child	Male
N15	36	Science and technology researcher	Master	First child	Male
N16	29	Institution staff	Master	First child	Male
N17	28	Institution staff	Undergraduate	First child	Female
N18	27	Nurse	Undergraduate	First child	Female

### 3.6 Data collection

#### 3.6.1 Intervention implementation

The researchers conducted education for pregnant women and their family members to obtain informed consent during the prenatal period. Mothers and infants who met the inclusion criteria would enter the SSC intervention process. First, the newborn would be placed on a dry towel prepared in the mother's abdomen after delivery, the midwife would dry the newborn within 30 s and assess the mother's and baby's condition to decide whether to carry out the intervention. The umbilical cord was then cut. Then, the researcher removed the towel and placed the newborn on the mother's bare chest and abdomen in a prone position for SSC, wearing only a diaper and cap. The newborn's head was tilted to one side, the back was covered with a blanket, the mother embraced the newborn with her hands, the delivery bed located in the mother's upper body was adjusted to an inclined position and the bed blocks on both sides were pulled up. Finally, after ensuring the safety of mother and baby, the SSC would continue.

Meanwhile, the circulating midwife would observe the condition of mothers and infants at all times. If the mothers and infants had abnormal conditions, the mother complained of discomfort such as palpitation and nausea, felt uncomfortable holding the newborns or the newborns had unstable skin colour or other abnormal phenomena. SSC should be terminated in time and could not be performed during the observation period in the delivery room. If mothers and newborns had no abnormal conditions, SSC was continued until the end of the postpartum observation period (2 h after delivery).

In this study, the mean duration of SSC was 92.82 ± 5.742 min, with the longest duration being 105 min and the shortest duration being 80 min.

#### 3.6.2 Semi‐structured interview

An interview outline is shown in Table 2, and a face‐to‐face semi‐structured interview method was adopted; iFlytek Recording App on the mobile phone was used for interview recording; the average time of interview was 19 ± 11.792 h after postpartum; and all interviews were conducted in the wards of puerperae to avoid interference from others, which lasted for about 20 min. The researcher wrote an interview note immediately after the interview.

**TABLE 2:** Semi‐structured interview outline of mother–infant skin‐to‐skin contact.

No.	Questions
1	How do you feel when the baby is lying prone in your bare chest and abdomen?
2	How would you describe this new life? What do you notice about your baby's reactions?
3	How do you feel differently about your baby's emotional status after skin‐to‐skin contact?
4	What effect does the skin‐to‐skin contact have on your transition to the mother's role?
5	Do you think whether skin‐to‐skin contact with your baby can help ease the discomfort of childbirth?
6	What is your overall feeling during the early skin‐to‐skin contact in the delivery room?
7	Are you willing to continue skin‐to‐skin contact when you get home?

#### 3.6.3 Recording and transcription data

After each interview, the iFlytek Recording App on the mobile phone was used to transcribe the interview, and the transcripts (including interview notes) were checked and revised one by one by playing back the recordings. A total of 18 transcripts had a size of about 615 KB /629,811 bytes; in order to ensure the accuracy of the interview content, the transcripts and audio files were sent to the parturient woman for verification.

### 3.7 Data analysis

The three‐level coding method of procedural grounded theory (Long et al., 1993) and the Graneheim & Lundman qualitative content analysis method (Giannantonio, 2010) were combined to code the interview content step by step and establish the core categories in NVivo 12 software (Feng, 2020). Two researchers coded and discussed a portion of the manuscript, with third‐party participation to verify and reach an agreement, and then agreed to develop a coding manual; coded the remaining documents according to the manual; and analysed qualitative data from a quantitative perspective using SPSS25.0 to verify the results of qualitative coding. Member checking and double testing to increase trustworthiness of the study results.

1. Primary coding: Called the first‐level coding, the proceduralized grounded theory was adopted to code the transcript sentence by sentence and extract the original concepts, and the primary coding was also known as open coding. First, the transcript was read through to get a sense of wholeness; according to the voice, flexible selective coding was done word by word, sentence by sentence or paragraph by paragraph, so as to extract the original concepts and form semantic units. Two methods such as new coding and in vivo coding were adopted, and the former was used to generalize and extract the participants' original words and to name, conceptualize and abstract the participants' words to form new nodes; the latter was used to directly cite participants' original words as nodes, that is, when the words used by the participants were infinitely close to the words to be abstracted by the coding technique, the participants' original words were cited. A total of 1142 original reference points were finally formed in this study. Based on the original reference points, the nodes with low frequency and deviation from the interview outline were deleted and repeated and similar nodes were merged, and then repeated comparison and classification of nodes were made to establish 60 primary coding categories (see Table 3).
2. Secondary coding: Secondary coding was also called spindle coding. The primary coding nodes were taken as the base points to perform a new round of comparison and reclassification, the codes with common characteristics were classified into the same category and finally, a total of 23 subcategories were extracted (see Table 3).
3. Core coding: The core coding is also known as selective coding, and the secondary coding categories were generalized to form the core categories. Five core categories were formed in this study (see Table 4; the items in bold were the core categories), and the core categories were linked together to form the themes of the study.

**TABLE 3:** Distribution and composition of primary categories and subcategories of interview transcripts.

No.	Primary categories	Reference point	Subcategories
1	Mood during pregnancy	12	Mood status during pregnancy
2	Birth experience	48	Birth experience before SSC
3	Effective for pain relief	32	SSC relieves labour pain
4	It still hurts to think about it	9	
5	Quiet	8	SSC is beneficial for recovery
6	Safe	3	
7	Perceived	6	
8	Restored	12	
9	Healing	15	
10	Calm	8	
11	Easy	14	
12	Worth	29	
13	Fulfil a task	21	Role transition before SSC
14	Not in role	10	
15	No desire to protect	2	
16	Sensitive to baby's needs	17	Role transition after SSC
17	Arouse the desire for protection	14	
18	Role identity – I am a mother	50	
19	No obvious role transition	3	
20	Stimulate milk secretion	2	Attitude towards breastfeeding after SSC
21	Rooting reflex	6	
22	Willing to breastfeed the baby	7	
23	Negative feelings before SSC	15	Feeling before SSC
24	Positive feelings before SSC	6	
25	Negative feelings during SSC	20	Feeling during SSC
26	Positive feelings during SSC	212	
27	Difference in feeling between lying on body and in mother's womb.	21	Difference between visual and tactile feelings
28	Difference in feeling between seeing and touching a baby	16	
29	Difference in feeling between direct SSC and lie separated by clothes	4	
30	Mothers love to interact with their babies	15	Mother's demand for touching baby
31	Try to get a clear look at the baby	15	Demand for touching baby
32	Actively touch the baby	13	
33	Have expectations for the baby	4	Maternal–foetal interaction
34	Baby in imagination	3	
35	Feel the foetal movement	10	
36	No sense of role substitution	17	Role positioning during pregnancy
37	Be aware of role substitution	17	
38	Affective state	12	Maternal–foetal attachment
39	Visual capture	4	Baby's characteristics before SSC
40	Hear the crying	3	
41	Observe the baby's features during SSC	98	Baby's characteristics during SSC
42	No sense of emotional connection	18	Mother–infant connection before SSC
43	Lack of emotional arousal	15	
44	Sense of alienation	10	
45	Sense of safety	24	Mother–infant connection after SSC
46	Sense of emotional connection	74	
47	Arouse the maternal love	47	
48	Sense of intimacy	23	
49	Sense of achievement	10	Change in parenting initiative
50	Sense of potency	16	
51	Sense of confidence	4	
52	Sense of responsibility	4	
53	Expectation	5	Expectation for guidance
54	Be good for babies	25	Impetus to continue SSC
55	Improve family relationships	6	
56	Meet the mother's needs	3	
57	Willing to adhere to SSC	16	Willing to continue SSC
58	Willing to accept direct bare SSC	5	
59	Understanding degree	3	Perception in SSC
60	Worry	10	

*Note*: The serial numbers 1–60 correspond to 60 primary codes, while the subcategories correspond vertically to 23 subcategories that correspond to at least 1 or more primary categories. The reference points refer to the number of codes and the frequency of occurrence of the coding, which is the exclusive term of the Nvivo12 software.

**TABLE 4:** Distribution of core categories and subcategories of interview transcripts.

Core categories (reference points)	Subcategories (reference points)
**Birth experience** (196)	Maternal emotional state during pregnancy (12)
	Mother's birth experience before SSC (48)
	SSC relieves labour pain (41)
	SSC is good for physical and mental recovery (95)
**Role transition** (132)	Mother's role transition before SSC (33)
	Mother's role transition after SSC (84)
	Attitude towards breastfeeding after SSC (15)
**Feeling about SSC** (294)	Mothers' feelings before SSC (21)
	Mothers' feelings during SSC (232)
	Difference feelings between visual and tactile (41)
**Mother–infant relationship** (422)	Mother's demand for touching baby (43)
	Maternal–foetal interaction(17)
	Role positioning during pregnancy (34)
	Maternal–foetal emotional state during pregnancy (12)
	Baby vital signs perceived by mothers before SSC (7)
	Baby physical characteristics perceived by mothers in SSC (98)
	Mother–infant emotional connection before SSC (43)
	Mother–infant emotional connection after SSC (168)
**Parental efficacy** (107)	Change in parenting initiative (34)
	Expectation for professional guidance (5)
	Impetus to continue SSC after returning home (34)
	Willing to continue SSC after returning home (21)
	Mother's perception of SSC (13)

*Note*: There are five core categories in bold, which contain 23 subcategories contents on the right side.

### 3.8 Ethical considerations

The study followed the principles of voluntariness and confidentiality, all participants had signed a consent form and all information would be kept confidential.

### 3.9 Rigour

The experiments and interviews were conducted by the researcher herself to ensure that the experimental procedures were conducted according to standards, and the interviews were conducted according to the interview outline. To minimize the research error, the researcher is a midwife with professional psychological learning experience who is familiar with the SSC process and has interviewing skills. The researcher had no SSC experience during her childbirth; regret has become one of the research motivations. To avoid the impact of researcher's sentiment on the objectivity of the results, each interview has notes to distinguish researcher's idea from the core content of the interview. Another researcher is a psychology professor with rich experience in qualitative research and strictly controls the research process and ensures the objectivity of the research.

## 4 FINDINGS

### 4.1 SSC improves the childbirth experience of puerpera

#### 4.1.1 Childbirth experience of puerpera before SSC

This category included three subcategories such as emotional state during pregnancy, birth experiences before SCC and birth experiences after SCC. During pregnancy, due to factors such as worries about the health of the foetus and fear of childbirth, the maternal mood during pregnancy usually is focused on curiosity, anxiety and fear. However, when the newborn is born, the mother has richer experiences, including weight relief, anxiety, nervousness, fatigue and uncomfortable feelings. However, the experiences at that moment were mostly negative.

#### 4.1.2 Childbirth experience of the puerpera after SSC

After the delivery of the newborns, the puerpera had to undergo a series of operations such as delivery of the placenta and suturing of the wound, so they were mostly focused on their own discomfort and pain. When the puerperae touched their newborns, they were distracted from the pain and thus could tolerate the pain during wound suturing; they were immersed in the joy of new life and could perceive the baby in a quiet environment, which facilitated the puerpera's recovery so that they could feel relaxed, calm, secure and feel that all the hardships were worth it. SSC could reduce the pain of the puerpera and play a healing role in their physical and mental recovery, so as to make the childbirth experience positive and beautiful. N6: ‘When the baby is lying prone on my body, I feel that the pain is bearable. When my attention is on perceiving its life, I do not pay much attention to the pain in the lower body’. N5: ‘Since then, everything has disappeared and healed, and I think it is worth it’.

### 4.2 SSC contributes to role transition of puerpera

#### 4.2.1 Role and status of puerpera before and after SSC

SSC could speed up the process of role transition for the puerpera. They said that before SSC, their job was done after delivering the baby, they could only perceive the newborn by seeing and hearing, they still had no real feelings for the child and they had not yet developed a desire to protect the child. After SSC, the feeling of being a mother came immediately, with a strong sense of role substitution, which aroused her desire to protect the baby unconditionally; and the mother became sensitive to her baby's demand signals. N3: ‘The feeling of being a mother is more three‐dimensional than that when the baby was sleeping in my womb, and I feel that I am going to be a real mother’. N17: ‘I had no feeling of wanting to protect my child before, but the feeling of wanting to protect him suddenly comes out after accepting SSC’.

#### 4.2.2 SSC changes the attitude towards breastfeeding

The puerpera received the lactation signal in her body during SSC, and the newborn had a rooting reflex, which helped the mother to establish her confidence in breastfeeding. N1: ‘Sometimes when I'm holding my baby, I feel a kind of recovery, and I start to feel the milk secretion in my breasts, and sometimes I even feel the milk leaking out. I think this is a very intuitive and obvious effect of SSC on my body’.

### 4.3 SSC increases the positive feeling of puerpera

#### 4.3.1 Feelings of puerpera before SSC

Before SSC, the childbirth experience of puerpera was relatively simple and their feelings about newborns were abstract; the positive feelings included pleasant surprise, peace of mind and intuition. Negative feelings included unkindness, untruthfulness, lack of feeling and sense of alienation. The puerpera talked about the differences in feeling between seeing and touching the baby, including the difference in feeling between the baby lying prone on the mother's body (without clothes) and sleeping in the mother's womb, the difference in feeling between seeing and touching a baby and the obvious difference in feeling between direct SSC and SSC separated by clothes. N1: ‘When I have no contact with my baby, I still feel a little alienated; once I hug him… I feel that I am a mother during SSC, so I want to get close to him and take on that role’. N7: ‘The feeling of direct SSC is definitely not the same as when the baby sits on my chest with clothes to separate the skin, but the specific kind of feeling cannot be said; when my baby lies prone on my body(without clothes), I will feel the overflowing of maternal love’.

#### 4.3.2 Feelings of puerpera during SSC

The feelings of the mothers became rich during SSC and some mothers showed discomfort at the first contact with their newborns, including three negative feelings: fear, tension and rejection. N17: ‘when I started to hold the baby yesterday, I was still a little bit unaccustomed to the SSC, and I felt very strange and scared’. N8: ‘I was still a bit confused and nervous when I came into contact with my baby for the first time’. N10: ‘I felt very tired at that time, he was also very heavy, and it was so long for me to hold him for an hour’. N17: ‘At the beginning of SSC, I was just scared and had a feeling of rejection because I couldn't move at that time. If I could move, I would definitely run away’. Although some mothers had certain negative feelings at the beginning of SSC with their babies, they reported more positive feelings over time, including 29 types of positive feelings such as peace of mind, relaxed, touched, magical, comfortable, warm and happy. The close SSC with newborns creates a comfortable physical and mental environment for the parturient women and helps them maintain a good psychological state. N17: ‘This kind of warm feeling is very comfortable, my baby is also very calm and stops crying, he is lying on his stomach like this, and I feel a little empty at the moment when he is carried away in the middle period. When he is lying prone on my body, I am also getting used to him, and it is a little too exciting…’ N8: ‘Wow, it's amazing to have such a big baby coming out of my belly, just lying prone on my belly, I really feel that it's worth all this antenatal pain, and nothing is happier or better than that’. N6: ‘When my baby first came out, I felt his body temperature was a little lower than mine. When he was lying face down on my body, I felt his little heart moving on my body, when his little hands, feet and his skin touched my skin, that kind of soft and waxy feeling was so good’.

### 4.4 SSC strengthens mother–infant connection

#### 4.4.1 Mother–infant interaction during SSC

The puerpera said that they had conscious role substitution during pregnancy, but their role positioning was vague and one sided; they would have much expectation and imagination of the babies through the foetal movement and mother–infant interaction; and they thought that the babies were just a part of their bodies and thus had no special feelings. However, when the puerpera could have all‐round contact with the baby, the real perception of life signs such as heartbeat, breathing and temperature, as well as physical characteristics of limbs, head, weight and skin, would arouse their demands to interact with the baby, clearly see the baby and actively touch the baby, since then a mother–infant connection began to be established physically. The researcher observed that the mother touched and patted the baby from time to time to make a physical response to the baby's reaction during the SSC, and when the baby was lying on the mother's chest, there were movements such as raising the head, sticking out the tongue, rubbing the body upward and behaving as if looking for something between the breasts. SSC provided a platform for the mother and baby to have more physical contact… N5: ‘The feeling just came from the data on the report during the labor examination, thus I didn't have any feeling of love. When the baby comes out and lies directly on my body, a natural human emotion occurs, with a little more maternal love’. N3: ‘A physical connection appears after the baby comes out, a real life comes out of my body; compared to the one in my belly that I couldn't touch, this feeling is very different, that is, the baby can actually come out to interact with me’.

#### 4.4.2 Changes in mother–infant connection before and after SSC

This category included four subcategories such as sense of security, sense of connection, arousal of maternal love and sense of intimacy. Some puerperae said that before SSC they had a sense of alienation from the baby, the feeling towards the baby was unfriendly and unfamiliar, with a sense of distance; they had not yet developed a sense of mother–infant connection, and thus said that the child was someone else's and had nothing to do with them; the part of maternal love in the mother was not aroused; and the mother still had a more rational part. After SSC, a safe environment was created for newborns from a physical and psychological point of view, which helped them to adapt more quickly to the extrauterine environment and to be easily soothed; when a puerpera really perceived the body of their newborn and her feeling of being a mother became real, she psychologically accepted this newborn as her own child, a mother–infant bonding relationship was established, her feelings for the baby were aroused, the maternal love was released and she began to like the baby more and more. N2: ‘What I remember most is the difference between the feeling that the child was someone else's before it lay on my body and the feeling that I was connected to the child after I touched it. This is my son, so the part of motherly love comes out in me’.

### 4.5 SSC improves parental efficacy of puerpera

#### 4.5.1 Changes in parental initiative

The sense of accomplishment, strength, self‐confidence and responsibility of the mothers to be in caring for their children was enhanced by the SSC. N1: ‘I am immediately invigorated by the baby's movements. Sometimes when the maternity nurse is unable to soothe the baby, I can hug and soothe my baby, I feel a sense of accomplishment and am very happy’. N12: ‘I feel like I have another identity. My feeling about the baby was abstract before, but when I touch and perceive my baby, I feel more powerful and think that I have the closest person in the world’.

#### 4.5.2 Impetus and willingness to continue SSC

Of 18 respondents, 15 were willing to continue SSC after returning home, and the reasons included SSC was good for babies, conducive to building family relationships and met mothers' needs. Some puerperae also said that they had seen the introduction of SSC on TV and had not learned it specifically, but they were still very worried when the baby was lying prone on their bodies and therefore needed professional guidance. N12: ‘I can feel some happiness in this process and also want to promote emotional sublimation between my baby and me’. N6: ‘I think SSC can improve the relationship between husband and wife, promote the parent–child relationship and create a happy family atmosphere, and I want my baby to have SSC with his brother’.

### 4.6 Coding results of interview notes – SSC improves happiness in puerpera

All 18 interview notes were coded, the coding method was the same as the interview text coding procedure in the previous paragraph, the primary codes of the interview notes included 169 reference points and then the primary codes were repeatedly compared and classified into 20 secondary codes and finally, five core categories were obtained (see Table 5; the items in bold are the core categories).

**TABLE 5:** Distributions of subcategories and core categories of interview notes.

No.	Subcategories	Core categories (reference points)
1	Deep feelings	**Mothers' body language during the interview (17)**
2	Immersive enjoyment	
3	Smile on face	
4	Shining with tears	
5	Overflowing with happiness	
6	Trigger feelings faster	**Feeling about SSC in multipara (71)**
7	Move into role faster	
8	More obvious sense of pain relief	
9	More intense feeling	
12	More willing to breastfeed the baby	**New perspective of SSC (19)**
13	Younger mothers are not willing to accept long‐term SSC	
14	Change in the mother's acceptance of her baby	
15	Continue SSC to meet mother's needs after returning home	
16	Maternal status and background affect the acceptability	
17	Application of strap for SSC	**Implement a new strategy (6)**
18	Enrich the forms of health education	
19	Offer practical operation classes	
20	Moved	**Feelings of researchers (22)**

*Note*: There are five core categories in bold, which include subcategory contents on the left side, reference points refer to the number of codes and the frequency of coding occurrence, which is the exclusive term of Nvivo12 software.

#### 4.6.1 Happiness expressed by body language

There were five subcategories such as deep feelings, immersive enjoyment, smile on the face, shining with tears and overflowing with happiness. Some women smiled throughout the interview and seemed to be immersed in the previous scene of intimate contact with their babies. Some mothers had tears in their eyes when recalling the past, and the body language of the mothers revealed the happiness brought by SSC. The authors felt the happiness of the mothers during the interview and were touched several times, which also became the biggest motivation for the authors to continue studying SSC.

#### 4.6.2 Deeper feeling of multipara

Three multiparas in the interview always compared the newborn with the first child; the experiences at that time helped them to awaken their feelings, to get into the role faster and to have a more obvious sense of pain relief, a higher degree of recognition, a more intense feeling and a more obvious healing effect. N6: ‘One of the things I remember most about my baby lying on my body was the feeling of crying with joy… When I was pregnant with my first child, it felt just like that a job was done. Spring blossoms was another word that came to mind, all the happiness came, the sun was shining brightly, just like yesterday's weather’.

#### 4.6.3 New findings of the study

The mothers with SSC experience were willing to breastfeed their babies and said that the intimate contact could also meet their own needs and change their acceptance of the baby, and thus, they had the courage to be mothers; four mothers said that they could not see the babies clearly when the babies were lying prone on their bodies, which suggested the SSC position should be improved; and the awareness and concern about SSC among puerperae suggested that it is necessary to strengthen prenatal health education. Maternal education can be carried out throughout the whole perinatal period. During pregnancy, various courses can be opened to encourage new parents to attend the course together – theoretical and practical classes can be set up; theoretical classes can use videos, pictures and theoretical explanations to teach the benefits and methods of SSC, and practical classes can use newborn models to practice the methods of SSC; during the delivery period, midwives can instruct new parents in the delivery room about the specific methods and explain the precautions. During the postpartum hospital observation period, the nurse in charge can track the degree of mastery of new parents and provide timely guidance on the specific practices of subsequent SSC, so as to facilitate the continuation and familiarity of SSC; during the postpartum follow‐up period, the nurse in charge can track the implementation of new parents, and even hospitals and community hospitals can establish a system of consulting parents for co‐management and community hospitals can visit the home for one‐no‐one guidance. Thus, the implementation of SSC is promoted by changing the way of perinatal education to help new parents really master the knowledge and skills.

### 4.7 Significant differences in the number of coding reference points between before and after SSC

There were significant differences in the number of coding reference points in five nodes in Table 6 between before and after SSC, which included positive feelings of the puerpera, physical characteristics of the newborn as perceived by the puerpera, mother–infant connection, role transition and birth experience. The number of coding reference points after SSC was significantly higher than before SSC. SSC enriched the experiences of the puerpera so that she could use rich language to express herself when talking about SSC during the interview.

**TABLE 6:** Comparison of the number of coding reference points before and after SSC.

Item	Positive feelings	Baby's physical characteristics	Mother–infant connection	Role transition	Birth experience
Before SSC	6	7	43	33	48
After SSC	212	98	168	84	136

## 5 DISCUSSION

### 5.1 SSC helps the puerpera get positive psychological experience

SSC can meet the physical and psychological needs of both mothers and infants. The puerpera can get active and positive feelings from SSC, thus having reduced pain perception during wound suturing and improved childbirth experience. On the one hand, puerpera's attention is distracted during SSC (Kollmann et al., 2017), and on the other hand, SSC can promote the release of a large amount of neurohormones in the mother's body, including oxytocin, which is an anti‐stress hormone that can help reduce postpartum anxiety and restore calm in the puerpera. Endorphins can produce a strong analgesic effect, relieve pressure and give people a sense of pleasure (Uvnäs‐Moberg, 1998). Some studies have also shown that SSC can promote the secretion of a‐amylase from the mother's pituitary gland, and thus increasing the antioxidant level in the mother to relieve postpartum tension and pain (Yuksel et al., 2016). In this study, the participants said that they paid less attention to the pain after the baby lay prone on their bodies, and said that it was all worth it; it was so nice to perceive their babies within a short distance, the mothers said that all the past was gone with the wind and their states were also restored. SSC can increase the connection among the oxytocin, prolactin and endorphin systems in the mothers' brain areas and promote the release of various hormones in an interactive manner, improve the mothers' birth experience, create a positive psychological state for them and thus lay a physiological foundation for a good mother–infant relationship. The birth experience and positive feelings will directly affect the mother–infant interaction and thus affect the establishment of the mother–infant relationship (Cho, 1990).

In terms of exploring the effect of the psychological feelings of SSC on puerpera, this study also has some limitations. Because the interview was conducted after childbirth, that is, after the implementation of the SSC intervention, the real feelings of puerpera before and after SSC and their cognition of SSC might be affected by the childbirth itself, and the reliability and validity of the interview results might also be affected to some extent. Therefore, in the expected future study, the interview time points should be strictly divided to exclude the effect of childbirth itself, or the population should be divided, for example, a control group will be set up for the interview, and mothers who did not have SSC with their infants will be interviewed. Alternatively, excluding multiparas, the interview concerns only first‐born mothers to avoid the effects of childbirth itself. In addition, the period between the end of SSC and the beginning of the interview, the mother's feelings become richer as she spends more time with the newborn compared to before SSC, which makes it difficult to distinguish that the feeling coding reference points become more necessarily due to SSC; therefore, this is a limitation of this study, and future studies may consider advancing the time point of the interview to start immediately after the end of SSC to minimize interference.

### 5.2 SSC stimulates maternal behaviour of puerpera

The naked newborns lie prone on their mothers' breasts, and their behaviours such as sucking fingers, climbing up, slapping or massaging breasts with hands, raising heads, gnawing, latching on to the breast and sucking, activate the release of endogenous oxytocin in mothers' bodies (Hoffman et al., 1984). Oxytocin system can regulate interpersonal relationships, improve interpersonal mutual trust and empathy ability, promote mother's recognition of infants and emotional empathy, evoke maternal behaviour and enhance mother's sensitivity to respond to her infants. A study has shown that mother's parental sensitivity can reduce infant's negative emotions and enhance the regulatory effect of oxytocin on mother–infant relationship (Jones & Sloan, 2018). In turn, the infant's negative emotions will damage the mother's emotional function and lead to less‐than‐ideal parental behaviour (Takubo et al., 2019). As an effective means of soothing newborns, SSC can reduce the number and duration of crying and increase the sleep time of newborns after birth, which is conducive to improving caregivers' sensitivity to infants' emotional expressions and care demands, and thus help them to actively respond to various stimuli (sounds, expressions, etc.) from infants (Gao et al., 2010). In this study, participants reported that they were more likely to pay attention to the infant's demand signal and respond positively after SSC, thus creating a virtuous cycle between the mother's positive response and the infant's positive emotion.

Oxytocin and its receptor gene may also affect the development of social adaptive skills in infants by improving mother–infant interaction, regulating empathy and reducing fear and anxiety (Wu & Su, 2012). In the study of Agudelo et al. (2020), it was confirmed that the newborns who received SSC were more likely to adapt to the extrauterine environment, and the participants of this study revealed that comforting the baby in this way gave them a sense of accomplishment in parenting and made them more willing to interact with their babies. As the first experience of a newborn after birth, SSC can keep the pressure felt by the newborn at the beginning of life at a low level and avoid the release of a large amount of cortisol in the process of environmental adaptation by influencing the conduction of the amygdala and its nerve bundles through sensory stimulation and neurohormones, which is conducive to maintaining a moderate arousal state of the brain. The excitability of the amygdala in the infant brain affects its responsiveness. The more the sympathetic nervous system is excited, the higher the excitability of the amygdala, which allows the infant to be in a stable state in the mother's arms to avoid high reactivity and excessive negative emotions due to overactivation of the sympathetic nervous system (Vittner et al., 2018). A sharp and appropriate response is a reflection of the mother's parental competence, which is conducive to improving perceived self‐efficacy and realizing self‐worth. To provide optimal care, the mother is first required to focus her attention on infant stimuli and make appropriate responses to these stimuli, that is, responding to infant stimuli is an important basis for the occurrence of parental behaviours (Stein et al., 2014).

During SSC, the mother's perception and response to the infant's stimuli promote the connection of parental brain networks. Oxytocin is involved in the activation and connection of the parental brain network in the mother and becomes the physiological lubricant for the establishment of the attachment relationship between mother and child (Zhang et al., 2019). According to the concept of parental brain, there is a set of neural circuits in the human brain that is very sensitive to parental behaviours and emotions. It is a brain region involved in motivation and reward processing that is activated in response to the infant's stimuli (Swain, 2011). By responding to the infants' stimuli, mothers will activate their brain's motivation and reward networks, including the ventral striatum and oxytocin‐related hypothalamic region, thus increasing their sensitivity to infants' stimuli and motivating maternal behaviours to actively interact with infants and produce positive parental behaviours (Caria et al., 2012). Therefore, SSC can mobilize the expression of maternal behaviours and enhance their parental sensitivity by activating the maternal oxytocin system, thus accelerating maternal role transition, improving parental efficacy and promoting the establishment of mother–infant bonding.

### 5.3 SSC regulates the mother–infant relationship through tactile pathways

As one of the senses first developed in infants, tactile sense is an important part of the interactive communication mode between mothers and infants and plays an important role in guiding infants' attention, regulating their arousal level, behavioural state and negative emotions, and reducing pain (Abu‐Zhaya et al., 2017). Infants' response to touch is developed in parent–child interaction, and there is also an interaction between parents' response to their infants' touch and parental behaviours. More attention and feedback from parents on infants' sensory responses are conducive to the development of parent–child relationships (Mammen et al., 2016). Multisensory communication between mothers and infants during SSC can mobilize mothers' maternal behaviours. The interaction between the touch stimulation of infants and the parental behaviours of mothers can mobilize the maternal behaviours of mothers, which is conducive to the establishment of mother–infant relationships. Before SSC, the newborn was taken by the midwife to the radiology department for preliminary treatment, such as drying the body, and then the pregnant woman did not have any contact with the newborn, the mother can only perceive her baby by seeing and hearing; however, after close SSC, the mother can perceive the baby by touching in an all‐round way, thus her love for the baby arises naturally and the mother–infant relationship becomes more realistic and three‐dimensional.

In the process of SSC, touch and smell can stimulate the maternal secretion of oxytocin and oxytocin can help to reduce the mother's anxiety level. Previous studies have confirmed this conclusion; for example, Karimi et al. (2013) showed that mothers in the SSC group had significantly less anxiety in caring for their infants at 3 months after birth than those in the control group, and the score of maternal–infant attachment in the SSC group was higher than that in the control group. At the same time, SSC can trigger the sucking reflex of the newborn, and the sound and touch of the infant can mobilize the mother in response to the infant's stimulation. The mother's positive response can promote the newborn's rooting reflex again, and the sucking and stimulating nipple can promote the mother's oxytocin secretion in the early postpartum period (Karimi et al., 2016).

Temporary separation of newborns from their mothers after birth has adverse physiological and psychological effects on them, most obviously it can reduce mother–infant interaction and is not conducive to the establishment of the mother–infant attachment relationship. According to basic attachment theory, the closer a mother can be to her newborn to meet her needs, the better it will be for the newborn to feel safe and explore the world with the mother's full attention. Mother–infant separation and a lack of maternal concern and interaction are not conducive to the establishment of a mother–infant attachment relationship. A study also showed the positive effects of SSC in reducing maternal anxiety and promoting maternal attachment (Karimi et al., 2009).

### 5.4 SSC promotion strategies need improvement

SSC has been included as 1 of 12 strategies to improve quality of care and maternal–newborn outcomes and has been incorporated into health policies and patient education materials (Salvador et al., 2016). Despite the growing evidence of the positive impact of the Baby‐Friendly Initiative (BFI) on breastfeeding and maternal–newborn outcomes, few studies have examined the barriers and facilitators to the implementation of baby‐friendly practices that can be used to improve the uptake of BFI at the local or country level (Semenic et al., 2012). However, this study has confirmed that the 90 min of SSC is feasible and includes some facilitating factors, such as avoiding interference during delivery as one person per room, family accompaniment during the postpartum observation period to reduce maternal loneliness and the type of midwives doing all the work in the delivery room without affecting work arrangements. These facilitating factors can serve as references for other institutions or countries.

In this study, mothers had a higher degree of recognition for SSC, and the happiness obtained from SSC could be revealed through their oral language and body language, which was beneficial to their physical and mental recovery, thus laying a solid foundation for the establishment of the mother–child relationship. During the interview, some mothers hoped to receive professional guidance and said that the current contact position was not convenient for mothers to get a good look at their babies' appearance. These aspects may also be barriers and indicate that the implementation of SSC needs to be improved at this stage. At the national level, political support for the implementation of the Baby‐Friendly Hospital Initiative (BFHI) supports the expansion of baby‐friendly hospitals. Ongoing quality assurance is essential, as is systematic (re)assessment of BFHI‐designated hospitals. Baby‐friendly hospitals should provide support that favours long‐term healthcare relationships throughout the perinatal period. These findings can help support and further enable the effective implementation of the BFHI worldwide (Walsh et al., 2023). For example, healthcare institutions should integrate SSC into routine postpartum maternal and child care programmes, provide strong support in terms of human and material resources, enrich the forms of pregnancy education, conduct practical operation training and strengthen the professional training of medical personnel to improve their practical operation skills. While mother's knowledge was significantly associated with SSC practice (Eyeberu et al., 2022), strengthening prenatal education for mothers is also a promoting measure. All these measures will be conducive to the promotion of SSC, promote the development of mother–infant relationships by increasing the level of neurohormones and truly open a ‘window’ in the sensitive period for the establishment of mother–infant relationships.

### 5.5 Recommendations for future nursing education and research

This study carried out coding for the interview notes, and the new perspectives provide an overview of effective strategies for the implementation of SSC in the future. Studies have investigated the correlation between knowledge, attitude and implementation of SSC among nurses and the implementation of SSC in the perinatal setting, and the results showed nurses with bachelor or master significantly more knowledgeable and skilled in implementing SSC compared to others (Al Mutair et al., 2023). Studies suggested that leadership and knowledgeable practitioners could initiate education and clinical training to improve nurses' knowledge and awareness of the effectiveness of SSC. In addition, initiatives and behaviours would be improved following increased knowledge and perceived value. Lack of knowledge factors such as reluctance of medical staff can also be reduced or eliminated after experiencing SSC (Abdulghani et al., 2020; Deng et al., 2018). The researcher of this study has received professional training and has become a motivation for successful implementation based on the topic. In fact, the training needs to be popularized in the whole maternal and child health team and even directly included in the university courses of obstetrics, paediatrics and nursing. Meanwhile, with the use of high‐tech software (Nvivo12) to promote the progress of nursing research, the research in SSC field can rely on high‐tech to become more convenient and deep. The research scope of SSC has been widely involved in physiology and psychology internationally (Linnér et al., 2020), but there is still insufficient research on the psychological perspective in China. This study has expanded the research scope of China from the psychological and relational perspective.

**FIGURE 2:**
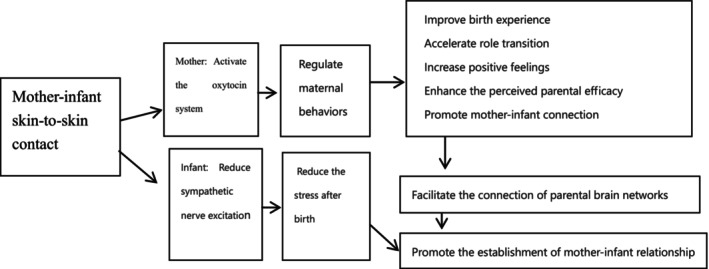
A theoretical model for the effect of SSC on mother–infant relationships.

By understanding the many benefits of SSC and strategies for implementation, healthcare providers can best support and promote this high‐quality, evidence‐based practice with mothers, newborns and their families (Hubbard & Gattman, 2017). The effects of continuous SSC on parental sleep quality and mood, as well as the quality of parent–infant interactions and salivary cortisol concentrations at discharge (Angelhoff et al., 2018), can be domesticated. Some mothers believe that the longer the SSC lasts, the more they will like their babies. The authors hope that in the future, we can develop a home‐based parent–infant SSC model to provide practical measures to facilitate the development of the parent–child relationship. Based on the existing studies at home and abroad and the results of this study, the authors establish a theoretical model for the effect of SSC on mother–infant relationship (Figure 2), which can be used as a theoretical basis for promoting SSC in maternal–infant healthcare institutions.

## 6 CONCLUSION

In this study, 18 interviewees received SSC for at least 1 h. SSC may primarily activate the oxytocin system to stimulate maternal behaviours in mothers, helping them to have a positive childbirth experience and faster role transition, improve perceived parental self‐efficacy and more quickly establish a mother–infant bonding.

SSC can coordinate and enhance the secretion of oxytocin in mothers, thereby triggering the output of their parental behaviours. Mothers respond to infant stimulation and express love to their infants in a timely manner, which makes infants feel safe and lays the foundation for the establishment of a secure attachment relationship between mothers and infants. The new infant stimuli generated during mother–infant interaction will have a positive feedback effect in stimulating the mother's parental sensitivity, creating a virtuous cycle. During SSC, mothers respond to infant stimuli such as sucking, scratching and touching; meanwhile, infants will engage in new activities such as crawling on mothers' breasts and making noises after perceiving mothers' responses; then, mothers will respond to these new activities again. This kind of cyclical, positive mother–infant interaction can lay the foundation for a good mother–infant relationship.

## AUTHOR CONTRIBUTIONS

All the authors have accepted responsibility for the entire content of this submitted manuscript and approved submission. Xiaoyan Feng and Yuqing Zhang designed the study methods, and Xiaoyan Feng collected data and analysis and wrote the manuscript. Yuqing Zhang modified the manuscript.

## FUNDING INFORMATION

The project name is Chongqing Key Specialized Construction "Clinical Nursing" Boutique Construction Project, the project No is 0203 [2023] No. 47 202336. There is no funding money.

## CONFLICT OF INTEREST STATEMENT

The authors have no conflicts of interest to disclose.

## APPROVAL ORGANIZATION AND PROTOCOL NUMBER INFORMATION

This research has been approved by Chongqing Key Specialized Construction ‘Clinical Nursing’ Boutique Construction Project 0203 [2023] No. 47202336.

## Data Availability

All data are true and reliableknow Wiley ‐ OAA policy.
